# Impact of Wheat Streak Mosaic Virus on Peroxisome Proliferation, Redox Reactions, and Resistance Responses in Wheat

**DOI:** 10.3390/ijms221910218

**Published:** 2021-09-23

**Authors:** Lidiya Mishchenko, Taras Nazarov, Alina Dunich, Ivan Mishchenko, Olga Ryshchakova, Ivan Motsnyi, Anna Dashchenko, Lidiya Bezkrovna, Yaroslav Fanin, Olga Molodchenkova, Andrei Smertenko

**Affiliations:** 1 Institute of Biology and Medicine, Educational and Scientific Center, Taras Shevchenko National University of Kyiv, 01601 Kyiv, Ukraine; korenevochka1983@ukr.net; 2 Institute of Biological Chemistry, Washington State University, Pullman, WA 991641, USA; taras.nazarov@wsu.edu; 3 Faculty of Agricultural Management, National University of Life and Environmental Sciences of Ukraine, 15 Heroyiv Oborony, 03041 Kyiv, Ukraine; iamishchenko@ukr.net (I.M.); dannaval@ukr.net (A.D.); 4 Laboratory of Plant Biochemistry, National Center of Seed and Cultivar Investigation, Plant Breeding & Genetics Institute, 65036 Odessa, Ukraine; olyaspring@ukr.net (O.R.); motsnyyii@gmail.com (I.M.); bezkrovnaya2015@ukr.net (L.B.); jaroslav-fanin@rambler.ru (Y.F.)

**Keywords:** peroxisome, virus, WSMV, redox homeostasis, yield

## Abstract

Although peroxisomes play an essential role in viral pathogenesis, and viruses are known to change peroxisome morphology, the role of genotype in the peroxisomal response to viruses remains poorly understood. Here, we analyzed the impact of wheat streak mosaic virus (WSMV) on the peroxisome proliferation in the context of pathogen response, redox homeostasis, and yield in two wheat cultivars, Patras and Pamir, in the field trials. We observed greater virus content and yield losses in Pamir than in Patras. Leaf chlorophyll and protein content measured at the beginning of flowering were also more sensitive to WSMV infection in Pamir. Patras responded to the WSMV infection by transcriptional up-regulation of the peroxisome fission genes *PEROXIN 11C* (*PEX11C*), *DYNAMIN RELATED PROTEIN 5B* (*DRP5B*), and *FISSION1A* (*FIS1A*), greater peroxisome abundance, and activation of pathogenesis-related proteins chitinase, and β-1,3-glucanase. Oppositely, in Pamir, WMSV infection suppressed transcription of peroxisome biogenesis genes and activity of chitinase and β-1,3-glucanase, and did not affect peroxisome abundance. Activity of ROS scavenging enzymes was higher in Patras than in Pamir. Thus, the impact of WMSV on peroxisome proliferation is genotype-specific and peroxisome abundance can be used as a proxy for the magnitude of plant immune response.

## 1. Introduction

Viruses cause major yield losses worldwide and represent a serious threat to food security. Although advances in the understanding plant–virus interactions lead to greater viral tolerance in crops, the evolution of viral virulence represents a constant challenge in agriculture. Keeping up with the pathogen-host arms race relies on the identification of novel tolerance traits and corresponding genetic markers. This, in turn, requires advances in understanding pathogen response on molecular, cellular, and organismal levels.

One of the common features of interaction between plant cell and viruses is the generation of reactive oxygen species (ROS) [1,2]. Rapid production of ROS during host–pathogen interactions is known as oxidative burst. The most important ROS are singlet oxygen (1O_2_), the hydroxyperoxyl radical (HO_2_·), the superoxide anion O^−^_2_, hydrogen peroxide (H_2_O_2_), and the hydroxyl radical (OH^−^). The bulk of ROS is generated in chloroplasts via the Mehler reaction, in mitochondria via electron transport, and in peroxisomes via photorespiration [3]. Some superoxide is produced in the apoplast by plasma membrane NADPH-oxidases. Superoxide is subsequently converted to hydrogen peroxide by superoxide dismutases. Other potential sources of apoplastic H_2_O_2_ include peroxidases and polyamine oxidases. Apoplastic H_2_O_2_ can permeate through the plasma membranes inside the cytosol.

Under normal conditions, ROS are the by-product of cellular metabolism and quickly neutralized by scavenging processes in, a so-called, redox homeostasis. Low concentrations of ROS in cells regulate metabolic processes and adaptation reactions. A greater rate of ROS production can overcome the cellular scavenging capacity resulting in rapid accumulation of ROS, oxidative stress, and oxidative damages to cellular components [1]. These oxidative damages are the main reason for yield losses [4]. During an incompatible interaction between a plant and a virus rapid ROS accumulation can induce hypersensitive response (HR), a type of programmed cell death that visually appears as necrotic lesions at infection site [1]. The HR restricts pathogen spread through the plant body.

Another important oxidant is nitrogen monoxide (NO). NO can activate the chain of free radical reactions or suppress them, as well as to be as a reducer and oxidant [5]. As a free radical, NO is highly reactive and can be transformed into other reactive nitrogen species (RNS) through nitrosylation and nitration. RNS can cause damages to cellular components in the same way as ROS, and affect properties of other molecules through nitrosylation and nitration processes [5,6].

Plants alleviate oxidative damages using ROS scavenging system consisting of two arms. The first arm includes enzymes like superoxide dismutase, catalase, ascorbate peroxidase, glutathione peroxidase, glutathione reductase, monodehydroascorbatereductase, and dehydroascorbatereductase. The second arm includes non-enzymatic antioxidants such as ascorbic acid, reduced glutathione, flavonoids, saccharides and others. The antioxidant enzymes can contribute to plant defense against viruses [1,7,8,9]. Thus, activity of ROS-scavenging system informs on tolerance to viruses.

Peroxisomes play an important role in redox homeostasis. One of the main enzymes of the photorespiration pathway, glycolate oxidase, inside peroxisomes is probably the main source of H_2_O_2_ in photosynthetic tissues. Here, the level of H_2_O_2_ production in peroxisomes is 2 times higher than in chloroplasts and 50 times higher than in mitochondria [10]. Peroxisomes also contain efficient ROS scavengers—catalase in their matrix and ascorbate peroxidase on the membrane [11]. Changes in metabolic activity, number, size, morphology, and rate of peroxisome movement differ between cell type, tissue, organism, stage of development, or environmental conditions [12]. Peroxisomes also contribute to generation of NO [6].

Peroxisomes play an active role in viral diseases [13]. For example, barley stripe mosaic virus inhibits ROS production in peroxisomes to bypass the host immune response [14]. Further, a multifunctional viral protein γb inhibits glycolate oxidase inside peroxisome which involved in the production of ROS. Other viral pathogens, e.g., Cucumber necrosis virus, cause accumulation of hydrogen peroxide in infected cells, potentially contributing to higher amount of peroxisomes in cells (peroxisome abundance) in response to the virus [15]. Tombusviruses convert peroxisomes into peroxisome-derived multivesicular bodies that facilitate the virion replication [16,17,18].

Peroxisomes can form *de novo* from the endoplasmic reticulum or through fission of existing peroxisomes [19,20]. Peroxisomal fission encompasses three phases; elongation, constriction, and fission [19,21]. Several proteins drive the fission: PEROXIN 11 (PEX11) promotes peroxisome elongation and tubulation [22,23]; DYNAMIN-RELATED PROTEIN 3 (DRP3), DYNAMIN-RELATED PROTEIN 5B (DRP5B) and FISSION 1 (FIS1) mediate the fission of peroxisomes [24,25].

A key mechanism of antiviral response in plants is the generation and cell-to-cell movement of small silencing RNAs (siRNAs), which targets viral genomes. Although, it has long been believed that this process is independent of peroxisomes, recent findings revealed that peanut clump virus (PCV) uses viral suppressor of RNA-silencing protein P15 to bind and transfer host siRNAs from the cytoplasm to peroxisomes, thereby contributing to suppression of plant immune system [26].

The plant innate immune pathway employs a two-level detection system, which involves plasma membrane-localized and intracellular immune receptors [27,28]. At the first level, the pathogen-triggered immunity is mediated by surface-localized pattern recognition receptors, which detect and recognize pathogen-associated molecular patterns or PAMPs [29]. The second level, effector-triggered immunity, involves intracellular immune receptors, designated as resistance proteins (R), which recognize—directly or indirectly—virulence effectors secreted by the pathogens into the host intracellular environment, thereby activating a defense response [27,28]. The immune responses downstream of the R proteins encompass ROS production, calcium ion influx, MAPK activation, salicylic acid accumulation and induction of genes associated with defense responses.

Amongst the transcriptionally up-regulated genes in response to pathogens are pathogenesis-related (PR) proteins. These proteins restrict the infection spread by accumulating in both infected and uninfected tissues. PR proteins contribute to the hypersensitive response and systemic acquired resistance against infection [30]. Two examples of PR proteins are β-1,3-glucanase (EC 3.2.1.39) and chitinase (EC 3.2.1.14). β-1,3-glucanase, also known as laminarinase, enhances fungal resistance in crop plants. These endoglucanase catalyzes the hydrolytic cleavage of (1,3)-β-d-glucosidic linkages in (1,3)-glucans and act primarily on glucans present in the fungal cell wall. The main substrate of chitinase is chitin, a natural homopolymer of β-1,4-linked *N*-acetyl glucosamine residues. Typically they are expressed constitutively at low levels in plant cell and accumulate in response to fungal, bacterial, viral infection [31,32].

Wheat streak mosaic virus (WSMV) is one of the most widespread and economically important viruses affecting wheat farming worldwide [33,34,35]. The impact of WSMV infection on redox homeostasis and peroxisome dynamics remains poorly understood and nothing is known about the impact of genotype on these processes. This study aims at determining how WSMV infection impacts on the redox homeostasis and on peroxisome proliferation in two winter wheat varieties Patras and Pamir in the field trials. Our results demonstrate that more robust ROS homeostasis and greater peroxisome proliferation in plants infected with WSMV are accompanied by lower yield losses.

## 2. Results

### 2.1. Comparison of Phenotypic Response to WSMV in var. Patras and Pamir

**Figure 1:**
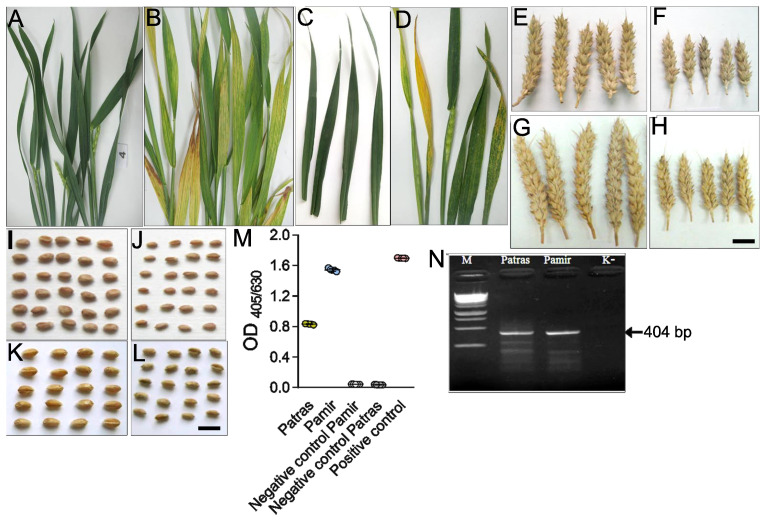
Diagnostics of WSMV infection in winter wheat leaves. (**A**,**B**) Representative images of healthy (**A**) and diseased (**B**) plants of variety Pamir. (**C**,**D**), Representative images of healthy (**C**) and diseased (**D**) plants of variety Patras. (**E**–**H**) Representative images of spikes collected from control (**E**,**G**) or infected (**F**,**H**) Pamir (**E**,**F**) or Patras (**G**,**H**) plants. Scale bar, 2 cm. (**I**–**L**), Representative images of spikes collected from control (**I**,**K**) or infected (**J**,**L**) Pamir (**I**,**J**) or Patras (**K**,**L**) plants. Scale bar, 1 cm. (**M**), Detection of WSMV in the infected leaves by ELISA. Total virus preparation was used as a positive control and a healthy-looking plants were used as a negative controls. Three technical replicates (individual plants) were performed. The difference between healthy and infected plants was significant (*p* < 0.001). (**N**), Detection of WSMV in the infected leaves using PCR. Gel electrophoresis shows 404 bp RT-PCR fragments amplified using RNA from infected leaves using primers for the viral coat protein gene. As a negative control, RNA isolated from a healthy-looking plant was used (lane marked as K). Lane marked as M contains DNA size markers corresponding to the following values: 10,000, 8000, 6000, 5000, 4000, 3500, 3000, 2500, 2000, 1500, 1000, 750, 500, 250 bp.

Survey of winter wheat fields in the Chernihiv region of Ukraine during May 2019 showed classical symptoms of viral infection in varieties Pamir and Patras. Specifically, abundant light green and yellow stripes spread on the leaf surface parallel to the main vein (Figure 1A–D). These symptoms are consistent with the WSMV infection.

This hypothesis was tested using ELISA with the antibody against WSMV. WSMV antigens were identified in the leaves of both Patras and Pamir with the disease symptoms, but not in the healthy-looking leaves (Figure 1E). Wheat plants were also tested by ELISA for the presence of barley yellow dwarf virus (BYDV), wheat dwarf virus (WDV), or brome mosaic virus (BrMV). Antigens of these viruses were not detected in the sampled material. ELISA data was confirmed by RT-PCR. WSMV-specific primers amplifying a 404 bp fragment of the WSMV coat protein gene were used. A band of the predicted size was amplified in the symptomatic samples but not in the healthy-looking control (Figure 1F). Several lower-size bands appear on the gel most likely as the consequence of non-specific primer binding to the viral template. These bands are missing in the negative control. The identify of the virus was confirmed using phylogenetic analysis with the published sequences of WSMV isolates (Supplementary Figure S1).

**Figure 2:**
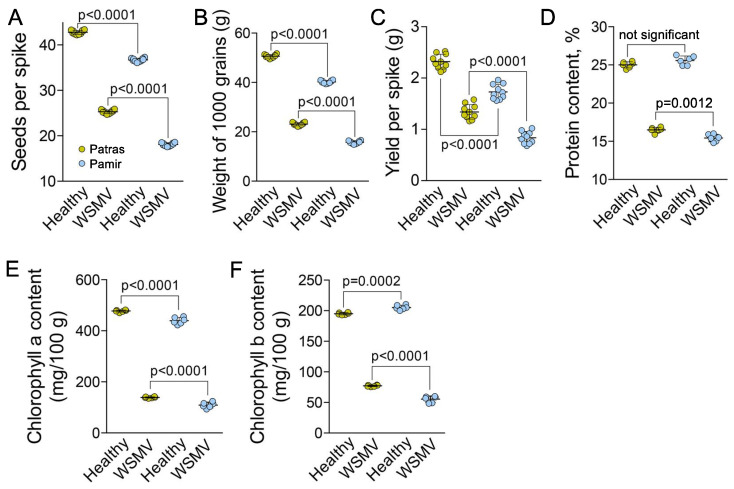
Impact of WSMV infection on yield and chlorophyll content. (**A**), Average seed number per spike in healthy and WSMV-infected plants. (**B**), Average weight of 1000 grains of healthy and WSMV-infected plants (**C**), Average weight of grains per spike in healthy and WSMV-infected plants (**D**), Relative protein content in leaves of healthy and WSMV-infected plants. (**E**,**F**), Chlorophyll *a* (**E**) and Chlorophyll *b* (**F**) content in leaves of health and WSMV-infected plants per 100 g of fresh leaf weight. Ten biological replicates (individual plants) were measured in **A** and six biological replicates (individual plants) were measured in (**D**–**F**). *p* values were calculated using non-paired *t*-test.

Yield analysis demonstrated that the infected plants of var. Patras exhibited 1.7 times reduction of seeds number per spike, 1.6 times reduction of 1000 grains weight, and 1.7 times reduction of seeds weight per spike relative to the healthy plants (Figure 2A–C). Var. Pamir is more sensitive to the infection with 2 times reduction of seeds number per spike, 1.9 reduction of 1000 grains weight, and 2.1 times reduction of seeds weight per spike (Figure 2A–C). Furthermore, the protein content in the leaves collected from WSMV-infected plants of var. Pamir was significantly reduced relative to that of var. Patras (Figure 2D).

It has been shown that compatible interaction between viral pathogen and the host plant induces senescence symptoms including degradation of photosynthetic pigments. As photosystem II provides significant part of ROS, inhibition of its activity reduced the magnitude of oxidative burst [36]. Thus, content of chlorophyll a and b informs on the ability of plant to mount the defense response [37,38]. Analysis of the content of chlorophyll a and b in the leaves demonstrated more significant reduction of both parameters in WSMV-infected Pamir (74.5% and 66% respectively) relative Patras (70.9% and 60.5% respectively; Figure 2E,F).

### 2.2. Impact of Viral Infection of Peroxisome Proliferation

**Figure 3:**
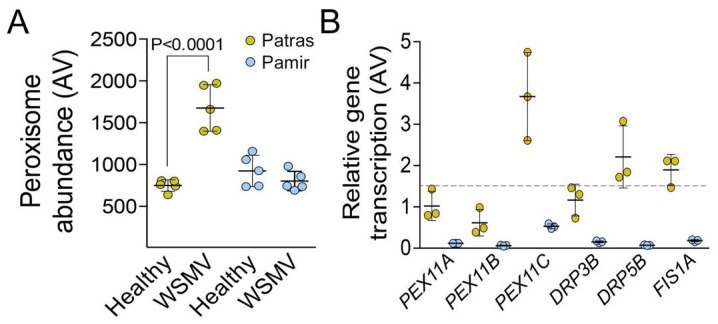
Impact of WSMV infection of peroxisome proliferation. (**A**), Peroxisome abundance in leaf of healthy and WSMV-infected plants. (**B**), Relative transcription level of genes responsible for peroxisome proliferation in leaf of healthy and WSMV-infected plants. The values are expressed relatively to the transcription level of the same gene in the control. The horizontal broken line represents 1.5 fold increase relatively to the control. *RLI* (RNase L inhibitor-like protein) gene was used as housekeeping control. Five biological replicates (individual plants) were analyzed in (**A**) and three individual plants were analyzed in (**B**). *p* values were calculated using non-paired *t*-test.

Peroxisome abundance was measured in total leaf extracts using our published procedure [39]. WSMV infection causes significant increase of peroxisomes abundance in Patras, whereas in Pamir the abundance remained unchanged (Figure 3A). Next, we measured transcription level of genes responsible for peroxisome proliferation, *PEX11A*, *PEX11B*, *PEX11C*, *DRP3A*, *DRP5B* and *FIS1A* using qRT-PCR. *RLI* (RNase L inhibitor-like protein) gene was used as housekeeping control. A change of 1.5 times or more relatively to the healthy control was considered as significant. We found that transcription level of *PEX11C*, *DRP5B* and *FIS1A* was significantly up-regulated in response to WSMV infection in var. Patras, whereas transcription of all peroxisome biogenesis genes was suppressed in var. Pamir (Figure 3B). It means that higher peroxisome abundance in var. Patras is accompanied by greater activity of peroxisome fission machinery.

### 2.3. Impact of WSMV Infection on ROS Stress

**Figure 4:**
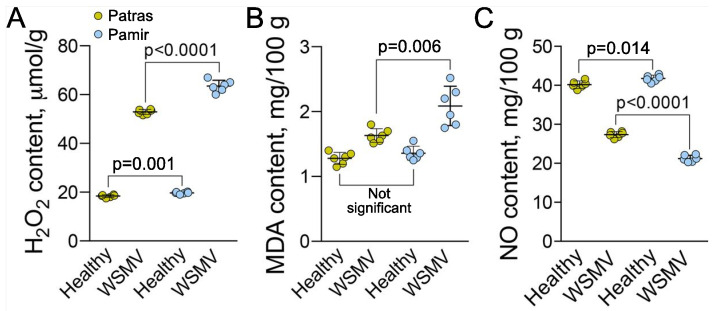
Analysis of oxidative stress in leaves of plants infected by WSMV. (**A**), Content of hydrogen peroxide per gram of leaf fresh weight in healthy and WSMV-infected plants. (**B**,**C**), Content of malondialdehyde (MDA; **B**) and nitrogen monoxide (NO; **C**) per 100 g of leaf fresh weight in healthy and WSMV-infected plants. Six biological replicates (individual plants) were measured per each treatment. *p* values were calculated using non-paired *t*-test.

A common plant response to pathogen invasion is oxidative burst that results in accumulation of ROS [1,40], peroxidation of membrane lipids, and accumulation of malondialdehyde (MDA) [41]. WSMV caused increase of H_2_O_2_ content in leaves of both varieties, but the value was greater in Pamir than in Patras (Figure 4A). Consistent with higher H_2_O_2_ content, the MDA content was also greater in Pamir than in Patras (increase by 49.1% and 32.1% respectively; Figure 4B). Content of NO in leaves following infection with WSMV decreased by 1.9 times relative to healthy plants in Pamir and by 1.5 times in Patras (Figure 4C).

**Figure 5:**
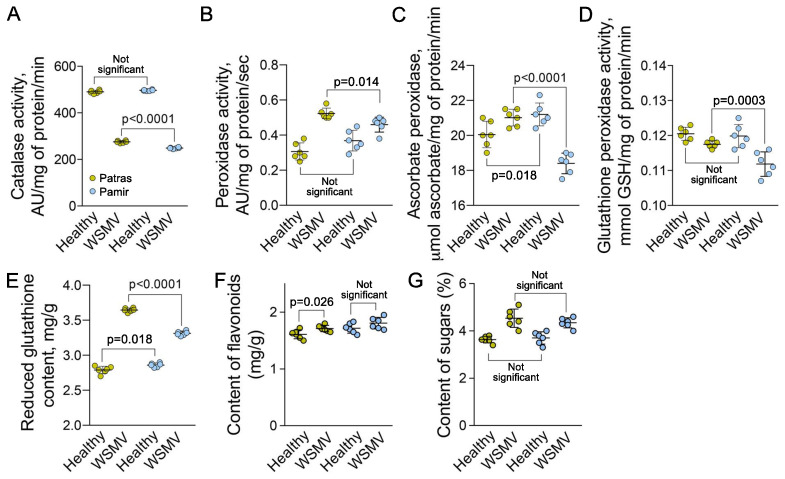
Response of ROS scavenging system to WSMV infection. (**A**–**D**), Activity of catalase (**A**); peroxidase (**B**); ascorbate peroxidase (**C**); and glutathione peroxidase (**D**) in leaves of healthy or WSMV-infected plants. (**E**,**F**), Content of reduced glutathione (**E**) and flavonoids (**F**) per one gram of leaf fresh weight from healthy or WSMV-infected plants. (**G**) Content of soluble sugars (monosaccharides and oligosaccharides) in leaves of healthy or WSMV-infected plants. Six biological replicates (individual plants) were measured per each treatment. *p* values were calculated using non-paired *t*-test.

Activity of ROS scavenging enzymes can be used as a proxy of ROS homeostasis. We measured four major enzymatic ROS scavengers, catalase, peroxidase, ascorbate peroxidase and glutathione peroxidase. The response of each enzyme differed. Catalase activity was reduced in both varieties in response to the infection (Figure 5A). Peroxidase activity in response to the infection increased in both varieties, but more in Patras than in Pamir (Figure 5B). Ascorbate peroxidase activity increases in Patras but decreased in Pamir (Figure 5C). Glutathione peroxidase activity was significantly lower in Pamir than in Patras (Figure 5D). We also determined impact of WSMV infection on content of several antioxidants. The content of reduced glutathione in leaves of WSMV-infected plants was significantly higher relative to healthy control in both varieties (Figure 5E). Content of flavonoids was not affected significantly (Figure 5F).

The decrease of catalase activity accompanied by the increase of H_2_O_2_ and MDA content indicates activation of oxidative processes in response to WSMV infection thus limiting virus replication and spreading to other cells. Transient suppression of catalase activity was reported during HR-type necrotic lesions in response to tobacco plants elicited by tobacco mosaic virus (TMV) and tobacco necrosis virus [42]. Activation of peroxidases and increase of reduced glutathione content in leaves infected by WSMV constitute protective reaction against the secondary oxidative stress caused by the virus [1,7].

Another important element of pathogen response are saccharides such as glucose, fructose, and sucrose [43]. Our results show that sugar content in leaves of WSMV-infected plants increased by 16% in Pamir and by 25% in Patras relative to the control (Figure 5G). However, the difference between the infected genotypes was not significant (*p* = 0.31)

**Figure 6:**
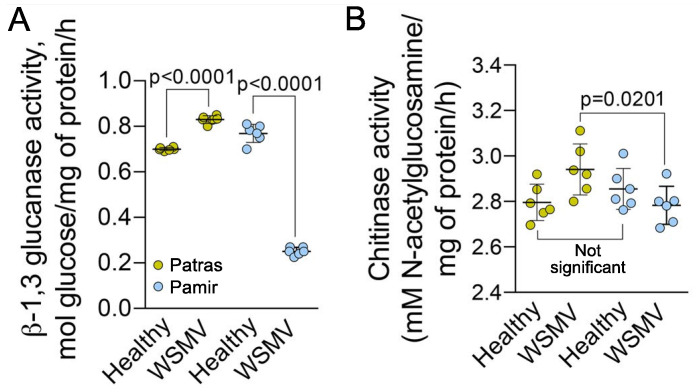
Impact of WSMV infection on activity of pathogenesis-related proteins. (**A**,**B**), Activity of β-1,3-glucanase (**A**) and chitinase (**B**) in leaves of healthy or WSMV-infected plants. Six biological replicates (individual plants) were measured per each treatment. *p* values were calculated using non-paired *t*-test.

Analysis of the pathogenesis-related proteins β-1,3-glucanase and chitinase showed significant differences between the varieties. Activity of β-1,3-glucanase increased in Patras, but reduced in Pamir in response to viral infection (Figure 6A); activity of chitinase was also higher in Patras than in Pamir (Figure 6B).

## 3. Discussion

### 3.1. WSMV Immunity and Redox Homeostasis

Redox homeostasis plays an essential role in plant immunity against viruses [44,45,46]. A significant increase of ROS content in leaf was reported in a susceptible genotype but not in a tolerant genotype of barley infected with barley yellow dwarf virus [47]. Spread of tobacco mosaic virus in tobacco plants is also accompanied by accumulation of ROS and MDA, and low activity or ROS scavenging system [48]. Accumulation of ROS and greater lipid peroxidation in var. Pamir relatively to var. Patras indicates inefficient intracellular ROS scavenging reactions in var. Pamir. The concentration of WSMV was greater in leaves of the var. Pamir than in var. Patras suggesting a weaker immune response.

Although, the immune response relies on the ROS scavenging system, there appears to be two types of response according to the activity of individual components. In one type, activity of catalase, peroxidase, SOD and glutathione reductase increase as was shown in *Phaseolus vulgaris* after inoculation with white clover mosaic potexvirus (WClMV) [49]. Infection with cucumber mosaic virus (CMV) also caused greater activity of catalase, peroxidase, and SOD in cucumbers, peppers, and tobacco plants, and greater activity of catalase, ascorbate peroxidase and SOD in cucumber and tomato plants [8].

Another type of response encompasses activation of peroxidases without activation of catalase. For example, CMV infection of *Arabidopsis* caused an increase in peroxidase and a decrease in catalase levels [9]. TMV and tomato mosaic virus (ToMV) induced a similar response in tomato and pepper [45]. Infection with plum pox virus (PPV) caused higher activity of peroxidase, ascorbate peroxidase, and dehydroascorbate reductase in a susceptible peach variety, and in the resistant variety higher activity of ascorbate peroxidase, monodehydroascorbate reductase, glutathione reductase, and SOD, whereas catalase activity was reduced in both varieties [50].

Both wheat genotypes analyzed in our study resemble the second type of response composed of higher peroxidase activity and lower catalase activity. The total peroxidase activity results from activity of many isoenzymes and only some of them play a role in the redox homeostasis. Other isoenzymes (e.g., class III isoperoxidases) contribute to different components of plant immunity such as strengthening cell wall or biosynthesis or the secondary metabolites [51,52,53]. As total peroxidase activity and reduced glutathione content in var. Patras were higher than in var. Pamir, both mechanisms are likely to play some role in reducing oxidative damages in the latter genotype. Increase of the ascorbate peroxidase activity in var. Patras and decrease of this activity in var. Pamir in response to WSMV infection suggest that ascorbate peroxidase is a key player in reducing oxidative damages.

The redox homeostasis is accompanied by the RNS homeostasis, though our knowledge about the function of RNS remains limited [5]. It has been shown that tobacco mosaic virus caused NO production in tomato plants during the first 12 h of infection [54]. The same virus increased activity of iNOS in leaves of resistant tobacco variety but not in the susceptible variety [55]. Experiments in *N. tabacum* and *A. thaliana* infected with tobacco mosaic virus demonstrated that NO is necessary for H_2_O_2_ accumulation, whereas NO generation was independent of H_2_O_2_. Furthermore, H_2_O_2_ appears to act downstream of NO to mediate induction of RNA Polymerase 1 (RDR1), which plays a critical role in RNA silencing for restricting systemic viral infection [56]. NO also acts downstream brassinosteroid-induced systemic resistance in *Arabidopsis* [57,58]. Down-regulation of NO in Pamir and Patras suggests NO signaling does not pay a role in WSMV immunity.

### 3.2. Peroxisome Proliferation Is a Component of Viral Immure Response

Many redox reactions occur in chloroplasts, peroxisomes, and mitochondria. Here we found genotype-specific peroxisome proliferation in response to WSMV. Proliferation of peroxisomes in var. Patras could be triggered by ROS through transcriptional activation of genes encoding peroxisome fission proteins PEX11C and DRP5B. Consistent with these observations, our previous electron microscopy analysis demonstrated enlargement and morphological aberrations of peroxisomes in WSMV-infected wheat leaf cells [59]. Oppositely, peroxisome abundance was not affected in leaves of WSMV infected var. Pamir. Furthermore, transcription of peroxisome fission genes was suppressed. Greater peroxisome abundance in leaves of cv. Patras suggests peroxisome proliferation is a component of plant immunity.

Reduction of catalase activity and an increase in the peroxisome proliferation could result in oxidative burst. It was noted that leaf lesions in *Datura stramonium* infected by tobacco mosaic virus contain cells with features of necrosis and also alive cells with higher number of peroxisomes relatively to that in the healthy cells [60]. H_2_O_2_ also act as the secondary messengers for inducing immune response in neighboring cells as well as a component of the hypersensitive response [45,49,61]. This hypothesis is supported by transcriptional up-regulation of genes encoding peroxisomal ROS scavenging enzymes and activity of these enzymes in response to pathogens [1,2,3,4,62]. The overall abundance of peroxisomes in the infected tissues could depend on the content of cells at different phases on the immune response. High peroxisome abundance indicates prevalence of cells with active immune response and slower virus spread.

### 3.3. Non-Oxidative Stress Related Elements of Plant Immunity

Chlorophyll content in leaves can be used to assess the immune response [63,64] as viral infection commonly causes reduction of both photosynthetic activity and chlorophyll content [63,65]. Reduction of chlorophyll *a* and *b* occurs in sugar cane leaves infected with sugarcane yellow leaf virus [66], wheat infected with BYDV [67], and cassava infected with cassava common mosaic virus [68]. Higher chlorophyll content indicates stronger immunity. For example, chlorophyll content was reduced in leaves of susceptible rice varieties by co-infection with rice tungro bacilliform virus and rice tungro spherical virus [37]. Leaf total protein content can also inform on the immune response. Analysis of 15 wheat genotypes showed that WMSV infection caused greater total leaf protein content in the tolerant genotypes but lower protein content in the susceptible genotypes [7,69]. In our studies, the chlorophyll content and the total leaf protein content was higher in Patras consistent with the higher tolerance to WSMV.

Another reason for the reduced chlorophyll content could be the inhibition of photosynthesis due to the accumulation of saccharides [70]. It has been shown that saccharide content increases in response to viral infection [66,70,71,72]. This effect can be a consequence of inhibiting phloem loading or slow phloem flow aimed at reducing the long-distance movement of viruses through the phloem [69]. Saccharides can also strengthen the immune response by inducing the accumulation of anthocyanin, activating PR genes, and/or functioning as antioxidants [67,73,74]. A similar increase of saccharides content in leaves of WSMV-infected Patras and Pamir indicates that both genotypes rely on this component for their immunity.

Activity β-1,3-glucanase and chitinase were greater in the WSMV infected plants of var. Patras than in var. Pamir. Glucanase activity has been shown to increase during viral infection in several species [75,76,77]. Expression of genes encoding β-1,3-glucanase increases in virus-infected *Arabidopsis* plants [78,79,80]. Glucanases are thought to contribute to viral spreading by regulating the size exclusion limit of the plasmodesmata [81]. The callose (β-1,3-glucan) deposited in the plasmodesmata neck region acts as a physical barrier for cell-to-cell movement of the viruses and glucanase deficiency delays the virus movement [82]. Glucanase was shown to be important for spreading but not for the multiplication of potato virus Y [81].

β-1,3-glucanase mRNA was detected in the lower inoculated leaves and upper uninfected leaves of tobacco plants on day 3 and 12, respectively, after inoculation of lower leaves with tobacco mosaic virus [83]. It means higher β-1,3-glucanase activity plays a role in systemic response [76]. The correlation between immunity and β-1,3-glucanase activity is not clear, as luteo- and poleroviruses caused up-regulation of genes encoding β-1,3-glucanase, and chitinase in both resistant and susceptible wheat varieties [84]. The increase of β-1,3-glucanase activity in var. Patras resembles a typical immune response to viral infection, whereas a significant decrease of this activity in var. Pamir suggests the immune response involves reduced plasmodesmata permeability.

## 4. Materials and Methods

### 4.1. Sampling

Winter wheat plants of varieties “Pamir” and “Patras” exhibiting streak mosaic symptoms were identified and collected during 2019 growth season in the fields in the Chernihiv region of Ukraine.

### 4.2. Enzyme-Linked Immunosorbent Assay

Double-antibody sandwich enzyme-linked immunosorbent assay (DAS-ELISA) was performed with total leaf extract using a wheat streak mosaic virus kit (Loewe, Sauerlach, Germany; catalogue number 07048S/500), barley yellow dwarf virus (BYDV; catalogue number 07076S), wheat dwarf virus (catalogue number 07082S), and brome mosaic virus (catalogue number 07016S). The analysis was performed on three replicates. The extracts were prepared by grinding 1–2 g of leaves in PBS buffer, pH 7.4, at the ratio 1 part of leaf to 2 parts of the buffer (weight to volume). Leaf samples from healthy-looking plants were included as negative controls. Positive controls were commercial protein preparations (Loewe, Sauerlach, Germany): WSMV (catalogue number 07048PC), BYDV (catalogue number 07076PC), WDV (catalogue number 07082PC), and BrMV (catalogue number 07016PC). The results were recorded on Thermo Labsystems Opsis MR reader (Thermo Fisher Scientific, Waltham, MA, USA) with Dynex Revelation Quicklink software at the wavelength of 405 nm. Samples were considered positive when their absorbance values at 405 nm were at least three times higher than those of negative controls [85].

### 4.3. RNA Extraction, RT-PCR and qRT-PCR

Total RNA was extracted from fresh leaves using an RNeasy Plant Mini kit (Qiagen, USA) following the manufacturer’s instructions. Two-step RT-PCR was performed. The cDNA was synthetized using RevertAid Reverse Transcriptase (MMuLV RT; Thermo Fisher Scientific, Waltham, MA, USA) according to the manufacturer’s instructions. Amplification was performed using thermocycler (Genetic Research Instrumentation Ltd., Essex, UK). WSMV-specific oligonucleotide primers for amplifying a fragment of WSMV coat protein genes were used (Table 1) [33]. The primers are designed to amplify the DNA product of 404 bp. Amplification was performed in 12.5 µL of Dream Taq PCR Master Mix 2× buffer (containing Dream Taq DNA polymerase, 2× Dream Taq buffer, 0.4 mM of each dNTP, and 4 mM of MgCl_2_), 7.5 µL nuclease-free water, 1 µL of each primer concentration 10 µM (final concentration 0.2 µM), and 3 µL of cDNA. The amplification reactions were set up as follows: initial denaturation for 3 min at 95 °C, followed by 35 cycles of 95 °C for 30 s, 60 °C for 30 s, and 72 °C for 30 s. The final extension was at 72 °C for 10 min. PCR products were separated on a 1.5% agarose gel with DNA markers SM 0311, Gene Ruler 1 kb DNA Ladder (Thermo Fisher Scientific, Waltham, MA, USA), stained with ethidium bromide, and visualized under UV light.

For qRT-PCR, total RNA was extracted from three individual plants (3 biological replicates). cDNA was synthesized using Maxima H Minus First Strand cDNA Synthesis Kit (Thermo Fisher Scientific, USA). The primers were designed to target all three homoeologs, as reported previously [86]. The primers are listed in Table 1. qRT-PCR reactions were performed using Fast SYBR™ GreenMaster Mix in 96-wells plates on ViiA 7 Real-Time PCR System with default ViiA™ 7 SYBR conditions (Thermo Fisher Scientific, MA, USA). Reactions were replicated three times and analyzed in QuantStudio™ Real-Time PCR Software v1.3. (Thermo Fisher Scientific, MA, USA), transcription levels were normalized to housekeeping gene RNase L inhibitor-like protein [87].

### 4.4. Biochemical Assays

Protein content was measured by the Kjeldahl method using automatic analyzer Kjeltec Auto-1030 (FOSS, Höganäs, Sweden) [88]. The total carbohydrate content was measured using the anthrone method [89]; 100 mg of leaf sample was added to 5 mL of boiling 2.5 N HCl and incubated for three hours in a boiling water bath. After the solution cooled down to room temperature, sodium carbonate powder was added to neutralize the acid until the effervescence ceased. The total volume of the reaction was adjusted to 100 mL, and samples were centrifuged at 5000 rpm for 15 min at 4 °C. Then 0.5 mL of the supernatant was mixed with 0.5 mL of distilled water and 4 mL of anthrone reagent. The mixture was heated for 8 min in a boiling water bath, rapidly cooled down, and absorbance of the reaction was measured at 630 nm in a UV-VIS spectrophotometer UVmini-1240 (Shimadzu, Kyoto, Japan).

Chlorophyll *a* and *b* content was determined using spectrophotometric method. One gram of fresh leaves was ground to a fine pulp using pestle and mortar in 20 mL of 80% chilled acetone. The material was centrifuged at 5000 rpm for five minutes at 4 °C, and the supernatant was transferred into a 100 mL volumetric flask. The debris was again ground in 20 mL of 80% chilled acetone, centrifuged as above, and the supernatant was transferred to the same volumetric flask. This procedure was repeated until the debris became colorless. The mortar and pestle were washed thoroughly with 80% acetone and the clear washings were collected in the volumetric flask. The volume was made up to 100 mL using 80% acetone. The absorbance values were read at 644 and 662 nm in a spectrophotometer against the blank, 80% acetone. Chlorophyll *a*, chlorophyll *b*, and total chlorophyll contents were calculated as milligram of chlorophyll per gram of leaf tissue using the following equations:

Chlorophyll *a*: [9.78 × (A 662) − 0.99 × (A 644)] × V × V_2_ × 100/w ×V_1_ × 1000

Chlorophyll *b*: [21.426 × (A 644) − 4.65 × (A 662)] × V × V_2_ × 100/w × V_1_ × 1000

where A = absorbance at specific wavelength, V = volume of the initial chlorophyll extract, V_1_ = volume of the initial chlorophyll extract, which was taken to dilute, V_2_ = volume of the diluted chlorophyll extract, V = and w = fresh weight of the tissue extracted [90].

Flavonoid content of the extracts was quantified using the aluminum chloride assay method [91]. The C-4 keto group and the hydroxyl group of either the C-3 or C-5 flavonoids react with aluminum chloride to form an acid-stable complex. In brief, 0.5 mL of plant extract was mixed with 1.5 mL of ethanol (95%) and 0.1 mL of 10% aluminum chloride; then 0.1 mL of 1 M sodium acetate was added and the final volume, adjusted to 5 mL with distilled water. After 30 min, the absorbance of the mixture was measured at 420 nm. Rutin was used as a standard. The flavonoid content of the plant extracts was expressed as milligrams of rutin equivalents per gram of leaf dry weight.  All the experiments were carried out in three replicates.

The activity of catalase was measured spectrophotometrically at room temperature by monitoring the decrease in absorbance at 240 nm resulting from the decomposition of H_2_O_2_. Catalase activity was measured according to the method of [92] in the reaction mixture containing 100 mM sodium phosphate buffer (pH 7.0), 30 mM H_2_O_2_, and 100 μL of crude extract in a total volume of 3.0 mL. One unit (U) of catalase activity will be defined as the amount of enzyme that caused absorbance change of 0.001 per min under assay conditions.

The peroxidase activity was measured using 4-methylcatechol as substrate. The increase of the absorption caused by oxidation of 4-methylcatechol by H_2_O_2_ was measured at 420 nm in the reaction mixture containing 100 mM sodium phosphate buffer (pH 7.0), 5 mM 4-methylcatechol, 5 mM H_2_O, and 500 μL of crude extract in a total volume of 3.0 mL at room temperature. One unit of enzyme activity will be defined as 0.001 change in absorbance per minute [93].

The glutathione peroxidase activity was measured by mixing 0.5 mL of plant extracts with 2 mL of Tris buffer, 0.1 mL of sodium azide, 0.2 mL of EDTA and. 0.2 mL of glutathione followed by 0.1 mL of hydrogen peroxide [94]. The reaction was mixed and incubated at 37 °C for 10 min. As a blank, we used a tube containing all the reagents except protein extract. The reaction was stopped after 10 min by the addition of 0.5 mL of 10% TCA. The samples were centrifuged and the supernatant was assayed for glutathione. The activities are expressed as μg GSH consumed/minute/mg protein.

Ascorbate peroxidase activity was measured by the rate of decrease in absorbance of ascorbate at 265 nm during ascorbate oxidation [95]. The assay was performed in a 3 mL quartz cuvette containing 0.5 mM ascorbate, 0.1 M phosphate buffer (pH 7.0), 1 mM H_2_O_2_. The blank contained all the above components, with the exception of H_2_O_2_. To 0.3 mL of protein extract, 0.7 mL of 0.5 mM ascorbic acid was added and the reaction was started by adding 0.1 mL of H_2_O_2_. A decrease in absorbance was recorded at 10 to 30 s intervals at 265 nm. One unit can be defined as one micromole ascorbate oxidized per min per mg of total protein in the extract. The content of reduce glutathione was measured using Ellman reagent (Sigma-Aldrich, Milwaukee, WI, USA) [96]. Lipid peroxidation level was determined using the tiobarbituric acid reactive substances MDA forms a 1:2 adduct with thiobarbituric acid (TBA) will be estimated spectrophotometrically A532 or fluorometrically [97]. NO content was measured by levels of nitric oxide stabile metabolites: NO_2_^−^ and NO_3_^−^ [98].

### 4.5. Measuring Peroxisome Abundance

Peroxisome abundance was measured using Nitro-BODIPY [39]. A 2 cm fragment of the leaf basal part was immersed in liquid nitrogen and ground to a fine powder using a pestle and mortar. Total leaf protein was extracted with 0.8 mL of the extraction buffer A (EBA; 20 mM Tris HCl, pH 7.4, 500 mM NaCl, 7M Urea). The debris was cleared by centrifugation at 3000× *g* for 30 min. Then 30 μL of the extract was mixed with 70 μL of freshly prepared 2 μM solution of N-BODIPY and 100 μL of water in 96-well plates and incubated for 10 min. The fluorescence intensity was measured at 490 nm excitation wavelength and 530 nm emission wavelength using Synergy Neo B spectrofluorometer (Biotek Instrument, Inc., Winooski, VT, USA). Five biological replicates (individual plants) with three technical replicates were performed per genotype and treatment. The background was measured as (i) 30 μL of each protein extract in 170 μL of water; and (ii) 30 μL of N-BODIPY supplemented with 170 μL of water per each 96-well plate. Both background values were subtracted from the N-BODIPY fluorescence signal value. The fluorescence intensity was normalized by the protein concentration measured with the Bradford Reagent (Biorad Laboratories, Hercules, CA, USA) using a calibration curve constructed with Bovine Serum Albumin. Fluorescence intensity was calculated in arbitrary units per 1 mg of protein.

### 4.6. Yield Analysis

Yield parameters were: seeds number per spike, weight of 1000 grains, and weight of grains per spike, measured in 10 plants per each experimental condition at maturity.

### 4.7. Statistical Analysis

Five biological repeats were conducted per each ELISA measurement. Each biological repeat contained leaves from five individual plants pooled together prior to grinding. Three technical repeats were conducted for each biological repeat. When appropriate, the technical repeats data were averaged to get the mean value for each biological repeat. DAS-ELISA was performed in three replicates4 biometric measurements, yield and its structure were performed in 10 replicates; all biochemical measurements were performed in six replicates. Data processing was performed using Microsoft Office Excel v.14.7.3 (Microsoft, Redmond, WA, USA) or Prism Graph 6 (GraphPad Software, San Diego, CA, USA). The differences between the mean values of the traits were evaluated by the Student’s *t*-test.

## 5. Conclusions

Our research addresses the proliferation of peroxisomes in the context of plant immune response to WSMV using two wheat varieties. Significant yield losses caused by the WSMV indicate that in our cropping system, both varieties are susceptible. However, the relative yield losses and virus spread in var. Patras were lower than in var. Pamir, suggesting higher tolerance in var. Patras. Peroxisome abundance was greater in the WMSV-infected leaves of var. Patras than in Pamir whereas in healthy plants, no significant differences were detected between the verities. It means peroxisomes play an important role in immunity to viruses and act as cellular markers of viral tolerance. Their role appears to be independent of catalase activity, but is likely to be related to the production of ROS for the oxidative burst. It means peroxisome abundance and peroxisome proliferation genes can be used as markers of plant immune response.

## Figures and Tables

**Table 1:** Sequences of primers used in this work.

Gene Name	Forward Primer	Reverse Primer
WSMV	TGCGGAACTTATCGACAACA	AATCACACGCTGCCACAATA
PEX11-A	CGCTAGGGGACGTGACTAA	CAGCGCCGACAGCAATC
PEX11-B	CAACCCGTTCTGCAACCAC	TTCCTATACCACCCAGCCCA
PEXX11-C	GAAGAACGCGATGCTGTCAA	TAAAAGGCAATCCTGCCAAG
DRP-3A	GACCTGCGGAGACAATGATAAC	GTTGGTCCTCTCGAAGATAGA
DRP-3B	TGGACGAGATACCGCTTGAA	CACTGAAAGGTTGTTGCTGC
FIS-1A	TCCAAGCAGACTGATGATGTG	TGGGCTGGTGGTTTTATCAAGA
Rnase L Inhibitor Like Protein (RLI)	CGATTCAGAGCAGCGTATTGTTG	AGTTGGTCGGGTCTCTTCTAAATG

## Data Availability

All published data is available on request.

## Supplementary Materials

Supplementary materials can be found at https://www.mdpi.com/article/10.3390/ijms221910218/s1.
