# Supplementary material for: Impact of Wheat Streak Mosaic Virus on Peroxisome Proliferation, Redox Reactions, and Resistance Responses in Wheat
Source: Int J Mol Sci. 2021 Sep 23;22(19):10218. doi: 10.3390/ijms221910218 (PMC8508189; doi:10.3390/ijms221910218)
Supplement: Supplementary file 1 [file ijms-22-10218-s001.zip › ijms-1349810-supplementary.pdf]

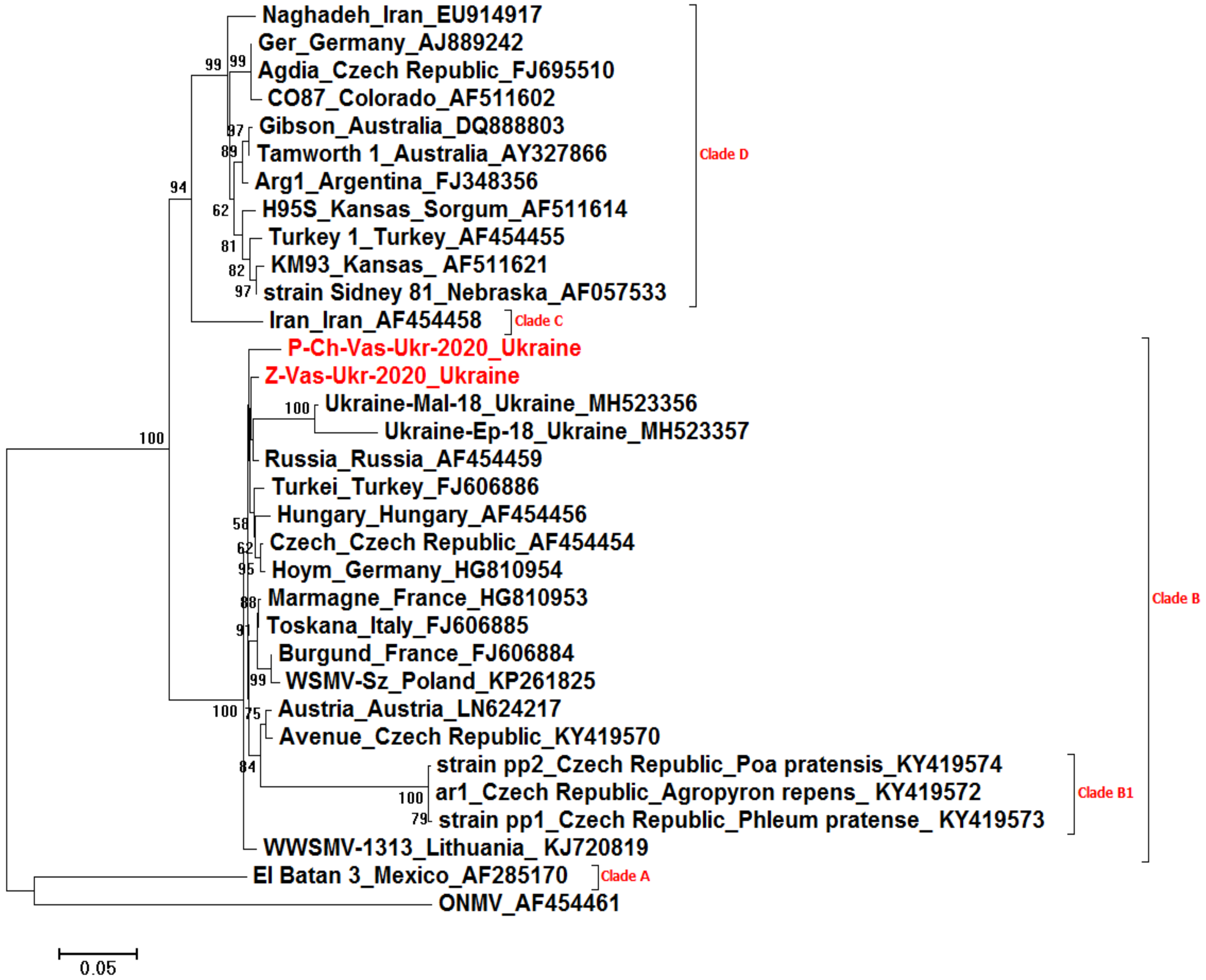


**Supplemental Figure S1.** Phylodendrogram of nucleotide sequences of WSMV isolates. Sequences isolated from wheat var. Patras (P-Ch-Vas-Ukr-2020) and from wheat var. Pamir (Z-Vas-Ukr-2020) are highlighted in red font. The tree was constructed using Neighbor-Joining method using nucleotide sequences of 676 bp coat protein gene region.
